# Supplementary material for: Mimivirus relatives in the Sargasso sea
Source: Virol J. 2005 Aug 16;2:62. doi: 10.1186/1743-422X-2-62 (PMC1215527; doi:10.1186/1743-422X-2-62)
Supplement: Additional file 1 — List of Mimivirus ORFs exhibiting a best match in the env-nr database [file 1743-422X-2-62-S1.pdf]

Mimivirus Relatives in the Sargasso Sea

Elodie Ghedin

Jean-Michel Claverie

List of Mimivirus ORFs exhibiting a best match in the env-nr database

Mimi ORF	Best Env-nr Match ID	Score	E-value	Best nr Match ID	Score	E-value
L18	gi|43710841|gb|EAF10508.1|	137.1	4.7e-31	gi|51244078|ref|YP_063962.1|	106.7	2.7e-21
L25	gi|42923128|gb|EAB28610.1|	169.1	5e-41	gi|34876677|ref|XP_214012.2|	163.3	1.1e-38
L75	gi|43124292|gb|EAC28017.1|	51.6	1.1e-05	gi|13794513|gb|AAK39888.1|	51.22	5.5e-05
L93	gi|42923128|gb|EAB28610.1|	177.9	1.6e-43	gi|40740451|gb|EAA59641.1|	156.8	1.5e-36
L100	gi|42923128|gb|EAB28610.1|	119	1.4e-25	gi|40740451|gb|EAA59641.1|	117.9	1.2e-24
L102	gi|44215848|gb|EAH75003.1|	46.98	0.00017	gi|19703853|ref|NP_603415.1|	41.2	0.035
R106	gi|43776260|gb|EAF42614.1|	139	8.1e-32	gi|37676542|ref|NP_936938.1|	107.1	1.4e-21
L111	gi|43296731|gb|EAD14168.1|	46.21	0.0006	gi|16805307|ref|NP_473335.1|	41.2	0.077
L113	gi|43770287|gb|EAF39597.1|	50.45	8.8e-06	gi|23498951|emb|CAD51029.1|	43.9	0.003
R118	gi|44281621|gb|EAI17665.1|	187.6	1.6e-46	gi|56965351|ref|YP_177083.1|	43.51	0.014
R132	gi|42849963|gb|EAA92307.1|	98.6	4.3e-20	gi|33864966|ref|NP_896525.1|	61.62	2.1e-08
R135	gi|44399015|gb|EAI99687.1|	99.37	1.4e-19	gi|48769830|ref|ZP_00274174.1|	97.06	2.7e-18
L136	gi|43375980|gb|EAD52870.1|	211.5	9.9e-54	gi|56708496|ref|YP_170392.1|	107.5	7.9e-22
R139	gi|42931622|gb|EAB32840.1|	57	1.8e-07	gi|30409752|gb|AAP32727.1|	55.45	2e-06
L143	gi|43788785|gb|EAF48931.1|	101.7	8e-21	gi|54031509|ref|ZP_00363643.1|	53.91	7.4e-06
L174	gi|43709436|gb|EAF09806.1|	48.52	0.0001	gi|13358136|ref|NP_078410.1|	45.44	0.003
L177	gi|43660484|gb|EAE85189.1|	47.75	0.00021	gi|56526423|emb|CAH77752.1|	44.67	0.007
L193	gi|43116621|gb|EAC24307.1|	138.7	1.7e-31	gi|59889773|emb|CAH19128.1|	110.2	2.6e-22
L206	gi|44511459|gb|EAJ77190.1|	210.7	2e-53	gi|13358409|ref|NP_078717.1|	163.7	1.1e-38
L207	gi|43582640|gb|EAE46140.1|	115.2	1.9e-24	gi|13358409|ref|NP_078717.1|	101.3	1.1e-19
L208	gi|43154232|gb|EAC42788.1|	54.3	6.8e-07	gi|23509124|ref|NP_701792.1|	44.67	0.002
L215	gi|43198765|gb|EAC64922.1|	45.44	9.8e-05	gi|30249486|ref|NP_841556.1|	43.9	0.001
R240	gi|43054398|gb|EAB93597.1|	83.57	9.3e-15			
L250	gi|43527692|gb|EAE18541.1|	133.7	3.8e-30	gi|17136758|ref|NP_476888.1|	121.3	7.9e-26
L251	gi|44049491|gb|EAG84927.1|	263.5	8.5e-69	gi|13357908|ref|NP_078182.1|	251.5	1.4e-64
R267	gi|42923128|gb|EAB28610.1|	114	2.9e-24	gi|40740517|gb|EAA59707.1|	111.7	5.7e-23
L279	gi|42977843|gb|EAB55869.1|	46.21	0.00082	gi|23508131|ref|NP_700801.1|	45.05	0.007
R296	gi|44416870|gb|EAJ11899.1|	138.7	6.8e-32	gi|48845024|ref|ZP_00299314.1|	132.9	1.5e-29
R299	gi|43651599|gb|EAE80654.1|	82.42	3.4e-15	gi|46440930|gb|EAL00231.1|	80.88	3.7e-14
L300	gi|44143264|gb|EAH36242.1|	59.31	1.5e-08	gi|50292185|ref|XP_448525.1|	54.3	1.7e-06
R301	gi|42942338|gb|EAB38176.1|	64.7	1.8e-09	gi|15042460|gb|AAK82240.1|	48.14	0.00068
L315	gi|42953235|gb|EAB43598.1|	88.97	5.4e-17	gi|56964473|ref|YP_176204.1|	70.86	5.9e-11

L318	gi|44268792|gb|EAI09019.1|	214.2	1.5e-54	gi|33416901|gb|AAH55597.1|	160.2	1e-37
L320	gi|43010993|gb|EAB72269.1|	101.3	8.6e-21	gi|30691953|ref|NP_174343.2|	67.01	6.9e-10
R322	gi|44500797|gb|EAJ70042.1|	230.7	1.1e-58	gi|1655695|emb|CAA93738.1|	160.2	7.5e-37
R325	gi|42880571|gb|EAB07497.1|	98.6	3.4e-20	gi|2738426|gb|AAB94453.1|	58.15	1.8e-07
R339	gi|43154902|gb|EAC43116.1|	60.46	7.5e-09	gi|17136888|ref|NP_476967.1|	58.92	7.6e-08
R341	gi|42929462|gb|EAB31721.1|	122.1	1.6e-26	gi|9628175|ref|NP_042761.1|	72.02	7.5e-11
R343	gi|43750296|gb|EAF29561.1|	131.3	2.3e-29	gi|30021937|ref|NP_833568.1|	129	4.6e-28
R354	gi|43821272|gb|EAF65062.1|	154.1	3.4e-36	gi|9631735|ref|NP_048514.1|	68.94	5.8e-10
R355	gi|43665722|gb|EAE87837.1|	121.3	1.1e-26	gi|450711|emb|CAA50819.1|	52.37	2.4e-05
L364	gi|42987215|gb|EAB60545.1|	72.02	1.7e-11	gi|4049749|gb|AAC97709.1|	63.54	2.4e-08
R366	gi|43171483|gb|EAC51308.1|	255	5.5e-66	gi|50302815|ref|XP_451344.1|	144.1	5.6e-32
R368	gi|43105861|gb|EAC18976.1|	113.6	4.3e-25	gi|13177431|gb|AAK14575.1|	73.94	1.1e-12
L371	gi|42973284|gb|EAB53609.1|	113.6	7.7e-24	gi|40556241|ref|NP_955326.1|	109.8	4.5e-22
L374	gi|43006294|gb|EAB69978.1|	58.15	6.6e-08	gi|49528793|emb|CAG62455.1|	37.35	0.452
L375	gi|44363842|gb|EAI74667.1|	131.3	1.3e-29	gi|450699|emb|CAA50807.1|	72.4	2.9e-11
L377	gi|43715140|gb|EAF12631.1|	141.7	4.2e-32	gi|37722439|gb|AAP33184.1|	127.9	2.6e-27
R378	gi|43174352|gb|EAC52755.1|	53.53	1.2e-06	gi|56489446|emb|CAI03544.1|	49.68	6.4e-05
R382	gi|44465210|gb|EAJ45951.1|	166	2.1e-39	gi|6319713|ref|NP_009795.1|	85.89	1.2e-14
R383	gi|43262281|gb|EAC96836.1|	69.32	6e-11	gi|17137638|ref|NP_477413.1|	51.6	5.1e-05
L388	gi|44253396|gb|EAH98417.1|	77.8	1.1e-13	gi|56961986|ref|YP_173708.1|	75.48	2.1e-12
L396	gi|43615322|gb|EAE62377.1|	173.3	5.1e-42	gi|13177345|gb|AAK14489.1|	167.5	1.1e-39
R398	gi|43115033|gb|EAC23498.1|	120.6	1.3e-26	gi|46226486|gb|EAK87480.1|	62.39	1.7e-08
R409	gi|43205815|gb|EAC68464.1|	52.37	2.4e-06	gi|37725924|gb|AAO38040.1|	38.89	0.097
R411	gi|43523940|gb|EAE16653.1|	78.57	1.8e-13	gi|3116125|emb|CAA18875.1|	77.8	1.2e-12
L417	gi|44253353|gb|EAH98387.1|	102.1	1.3e-20	gi|9632055|ref|NP_048844.1|	59.31	4e-07
R418	gi|44628310|gb|EAK59119.1|	122.9	4.8e-28	gi|11498373|ref|NP_069601.1|	121.3	5.8e-27
L425	gi|44004806|gb|EAG60442.1|	142.5	8e-33	gi|4587052|dbj|BAA76601.1|	119.8	2.2e-25
L426	gi|43035635|gb|EAB84418.1|	68.55	5e-11	gi|46241679|gb|AAS83064.1|	63.16	7.8e-09
R429	gi|43593604|gb|EAE51661.1|	216.5	4.1e-55	gi|9632061|ref|NP_048850.1|	152.5	2.9e-35
R430	gi|42973834|gb|EAB53885.1|	53.91	1.6e-06	gi|9632061|ref|NP_048850.1|	46.21	0.001
L432	gi|44173673|gb|EAH52299.1|	151.4	5.4e-36	gi|48730983|ref|ZP_00264729.1|	146.4	6.6e-34
R435	gi|43287470|gb|EAD09576.1|	66.63	4.8e-10	gi|23481897|gb|EAA18039.1|	48.52	0.00054
L437	gi|44215544|gb|EAH74836.1|	177.6	1.1e-43	gi|16151622|dbj|BAB69884.1|	157.1	6.1e-37
R439	gi|43111515|gb|EAC21807.1|	173.7	4.6e-42	gi|4587052|dbj|BAA76601.1|	164.5	1.1e-38
R440	gi|43011410|gb|EAB72486.1|	90.12	2.9e-16	gi|23510178|ref|NP_702844.1|	78.95	2.7e-12
R441	gi|43269509|gb|EAD00518.1|	209.9	4.8e-53	gi|3341805|gb|AAC27492.1|	137.9	9.4e-31
R443	gi|44226821|gb|EAH81210.1|	63.16	1e-09	gi|56470459|gb|EAL48116.1|	52.37	6e-06
R445	gi|43027882|gb|EAB80631.1|	50.06	4.1e-05	gi|34397716|gb|AAQ66777.1|	48.14	0.00061

L446	gi|42912213|gb|EAB23180.1|	95.52	7.2e-19	gi|47569527|ref|ZP_00240206.1|	84.73	5e-15
R447	gi|43125924|gb|EAC28806.1|	60.46	2.9e-09	gi|9632052|ref|NP_048841.1|	46.98	0.00014
R449	gi|43476579|gb|EAD93014.1|	122.9	1.1e-26	gi|13177377|gb|AAK14521.1|	62.39	6.9e-08
R450	gi|42883300|gb|EAB08831.1|	48.91	8.6e-05	gi|38683713|gb|AAR26889.1|	40.05	0.157
L451	gi|44145880|gb|EAH37686.1|	51.22	2.2e-05	gi|23612730|ref|NP_704269.1|	45.82	0.004
R453	gi|43138976|gb|EAC35259.1|	74.33	2e-12	gi|23481840|gb|EAA17997.1|	51.99	4.2e-05
L454	gi|43184473|gb|EAC57805.1|	72.79	2.7e-11	gi|16805082|ref|NP_473111.1|	70.09	7.1e-10
R468	gi|43479103|gb|EAD94286.1|	46.98	0.00017			
L471	gi|44521596|gb|EAJ83983.1|	48.91	0.0001	gi|23510142|ref|NP_702808.1|	44.67	0.008
R472	gi|43137277|gb|EAC34419.1|	111.7	7.2e-23	gi|33414605|gb|AAL38220.2|	63.54	9.3e-08
L479	gi|44350833|gb|EAI65324.1|	82.03	5.2e-15	gi|52788091|ref|YP_093919.1|	70.48	6e-11
L483	gi|42923128|gb|EAB28610.1|	161	1.9e-38	gi|42555731|gb|EAA78537.1|	157.5	8.3e-37
L485	gi|44073294|gb|EAG98118.1|	48.52	2.8e-05	gi|23509456|ref|NP_702123.1|	41.2	0.015
R489	gi|43155682|gb|EAC43508.1|	67.78	8e-11	gi|9631920|ref|NP_048709.1|	47.75	0.00032
L491	gi|44049493|gb|EAG84928.1|	61.62	7.8e-09	gi|56473336|gb|EAL50770.1|	48.52	0.00026
R493	gi|43155685|gb|EAC43510.1|	153.3	4.7e-36	gi|18726|emb|CAA39239.1|	88.58	5.7e-16
L496	gi|43924053|gb|EAG16876.1|	67.4	1.5e-10	gi|60468406|gb|EAL66411.1|	51.99	2.6e-05
R502	gi|44245809|gb|EAH92811.1|	46.21	0.00033	gi|23612359|ref|NP_703939.1|	38.12	0.343
L504	gi|43757624|gb|EAF33258.1|	51.22	2.1e-05	gi|23619172|ref|NP_705134.1|	40.05	0.192
L507	gi|44275242|gb|EAI13417.1|	99.37	3.3e-20	gi|9632038|ref|NP_048827.1|	88.97	1.7e-16
R508	gi|43850909|gb|EAF79670.1|	70.09	3.3e-11	gi|46440772|gb|EAL00074.1|	43.9	0.01
R512	gi|43084896|gb|EAC08655.1|	107.8	5.3e-23	gi|34333239|gb|AAQ64394.1|	68.55	1.3e-10
L539	gi|44612196|gb|EAK47432.1|	60.08	2.5e-08	gi|48095940|ref|XP_394563.1|	53.91	6.9e-06
R555	gi|44074387|gb|EAG98725.1|	221.1	6.6e-56	gi|48477311|ref|YP_023017.1|	129	1.4e-27
R568	gi|44444139|gb|EAJ31047.1|	82.03	2.2e-14	gi|42782567|ref|NP_979814.1|	78.18	1.3e-12
R569	gi|43851406|gb|EAF79912.1|	46.59	0.00037	gi|14247226|dbj|BAB57617.1|	42.36	0.027
R571	gi|42912213|gb|EAB23180.1|	103.2	2.9e-21	gi|54303145|ref|YP_133138.1|	87.43	6.5e-16
R592	gi|43246588|gb|EAC88951.1|	70.48	8.4e-11	gi|19173110|ref|NP_597661.1|	67.01	3.8e-09
L593	gi|44438438|gb|EAJ27151.1|	128.3	6e-29	gi|54029386|ref|ZP_00361528.1|	114.4	3.4e-24
R595	gi|43331658|gb|EAD31349.1|	97.44	1.6e-19	gi|42553185|gb|EAA76028.1|	90.12	9.8e-17
R596	gi|43382223|gb|EAD56003.1|	119.8	2.9e-26	gi|13177431|gb|AAK14575.1|	104.4	5e-21
R604	gi|43049485|gb|EAB91155.1|	47.37	0.00028	gi|23484531|gb|EAA19833.1|	44.28	0.01
L620	gi|42912213|gb|EAB23180.1|	57.77	2e-07	gi|19705025|ref|NP_602520.1|	55.45	3.9e-06
R640	gi|44283592|gb|EAI18988.1|	124.8	2.1e-27	gi|37519905|ref|NP_923282.1|	112.8	3.4e-23
R648	gi|44283592|gb|EAI18988.1|	167.5	2e-40	gi|37519905|ref|NP_923282.1|	122.1	3.8e-26
R654	gi|43291944|gb|EAD11806.1|	97.44	4.3e-19	gi|32263328|gb|AAP78373.1|	84.34	1.5e-14
R667	gi|43769283|gb|EAF39072.1|	55.07	2.1e-07	gi|52841308|ref|YP_095107.1|	50.83	9.6e-06
L687	gi|44566182|gb|EAK15053.1|	244.6	9.5e-64	gi|46097173|gb|EAK82406.1|	214.9	3.2e-54

R689	gi|44068613|gb|EAG95539.1|	150.6	1.2e-35	gi|24378003|gb|AAN59275.1|	144.1	4.4e-33
L690	gi|44283592|gb|EAI18988.1|	152.5	6e-36	gi|37519905|ref|NP_923282.1|	104.4	7.4e-21
R693	gi|43163235|gb|EAC47212.1|	119.4	8.9e-27	gi|49235486|ref|ZP_00329554.1|	115.5	3.2e-25
L716	gi|44016225|gb|EAG66729.1|	69.32	2.4e-11	gi|15921096|ref|NP_376765.1|	66.24	7.4e-10
R730	gi|44083879|gb|EAH03977.1|	199.5	4.8e-50	gi|6466376|gb|AAF12958.1|	123.2	1.7e-26
R753	gi|43500977|gb|EAE05248.1|	85.89	1.7e-15	gi|46447199|ref|YP_008564.1|	71.25	1.7e-10
R757	gi|43500977|gb|EAE05248.1|	73.17	9.5e-12	gi|46447096|ref|YP_008461.1|	65.86	6.2e-09
R758	gi|44662698|gb|EAK80724.1|	54.68	4.7e-07	gi|50755591|ref|XP_414809.1|	51.22	1.9e-05
R760	gi|43408903|gb|EAD65356.1|	97.44	3.4e-19	gi|20521133|dbj|BAA31672.2|	96.67	2.3e-18
R777	gi|42923128|gb|EAB28610.1|	95.52	6.3e-19	gi|18676694|dbj|BAB84999.1|	90.89	6.1e-17
L780	gi|43372155|gb|EAD50923.1|	280.4	1.3e-74	gi|56472969|gb|EAL50422.1|	237.7	3.7e-61
R791	gi|43884722|gb|EAF96312.1|	79.72	3.3e-14	gi|32419933|ref|XP_330410.1|	78.95	2.2e-13
R802	gi|44389434|gb|EAI92933.1|	59.69	2.7e-08	gi|25010522|ref|NP_734917.1|	57.77	3.9e-07
R815	gi|44467483|gb|EAJ47513.1|	109.4	9.5e-23	gi|52841586|ref|YP_095385.1|	107.8	1.1e-21
R832	gi|44335875|gb|EAI54742.1|	123.2	4.5e-27	gi|13475556|ref|NP_107120.1|	112.5	3.1e-23
R835	gi|43881322|gb|EAF94645.1|	87.43	1.8e-16	gi|24646968|ref|NP_650415.1|	84.34	5.8e-15
R837	gi|44398294|gb|EAI99192.1|	86.27	1e-15	gi|1845265|gb|AAB47805.1|	84.73	1.2e-14
R843	gi|44554385|gb|EAK06651.1|	53.53	2.9e-06			
R844	gi|43318044|gb|EAD24737.1|	83.57	1.3e-15	gi|53730512|ref|ZP_00151259.2|	76.64	6e-13
R846	gi|43713504|gb|EAF11838.1|	61.23	4.2e-09	gi|47214900|emb|CAG01031.1|	60.46	2.5e-08
R847	gi|44398294|gb|EAI99192.1|	74.71	2.4e-13	gi|28373837|pdb|1N0R|A	71.63	5.2e-12
R848	gi|42923128|gb|EAB28610.1|	110.5	1.5e-23	gi|58699292|ref|ZP_00374082.1|	98.98	1.7e-19
R850	gi|43710841|gb|EAF10508.1|	65.86	1.1e-09	gi|48834353|ref|ZP_00291368.1|	63.16	3e-08
R852	gi|43536538|gb|EAE22952.1|	115.9	2e-25	gi|33860982|ref|NP_892543.1|	99.37	7e-20
R853	gi|43166050|gb|EAC48610.1|	108.2	1.2e-23	gi|33860982|ref|NP_892543.1|	86.27	2.1e-16
R855	gi|44585905|gb|EAK28804.1|	120.6	2.4e-27	gi|33860982|ref|NP_892543.1|	101.3	6.2e-21
R877	gi|43313336|gb|EAD22405.1|	95.13	2.6e-19	gi|26990735|ref|NP_746160.1|	94.74	1.2e-18
L893	gi|44629372|gb|EAK59899.1|	62.77	1e-09	gi|57101548|ref|XP_541839.1|	59.31	2.7e-08
L894	gi|44600496|gb|EAK39086.1|	109.4	7.2e-23	gi|45684031|ref|ZP_00195462.1|	93.59	1.6e-17
R901	gi|42923128|gb|EAB28610.1|	162.5	5.5e-39	gi|42555731|gb|EAA78537.1|	157.1	9.1e-37
